# Supplementary material for: Multiparametric Analysis of Longitudinal Quantitative MRI Data to Identify Distinct Tumor Habitats in Preclinical Models of Breast Cancer
Source: Cancers (Basel). 2020 Jun 24;12(6):1682. doi: 10.3390/cancers12061682 (PMC7352623; doi:10.3390/cancers12061682)
Supplement: Supplementary file 1 [file cancers-12-01682-s001.pdf]

Supplementary Materials

Multiparametric Analysis of Longitudinal Quantitative MRI data to Identify Distinct Tumor Habitats in Preclinical Models of Breast Cancer

Anum K. Syed, Jennifer G. Whisenant, Stephanie L. Barnes, Anna G. Sorace and Thomas E. Yankeelov

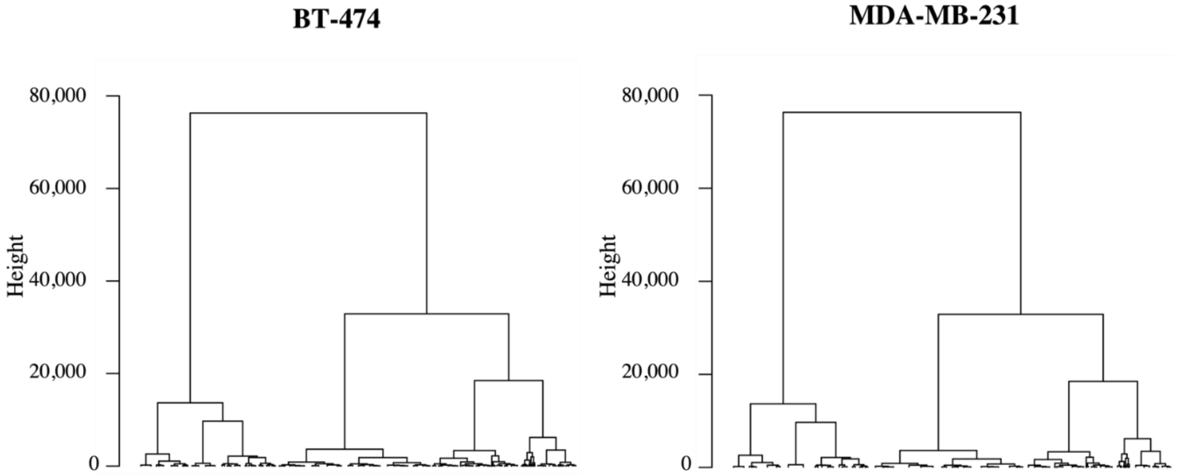

Figure S1. The resultant dendrograms from agglomerative clustering of BT-474 (left) and MDA-MB-231 (right) voxel data. A dendrogram shows the hierarchical relationship between voxel clusters (x-axis). The height of the branches (y-axis) indicate the order in which clusters were joined together, as well as the distance between clusters. Dendrogram branch height along with gap statistic plots were used to select a $k = 3$ for both BT-474 and MDA-MB-231 cohorts.

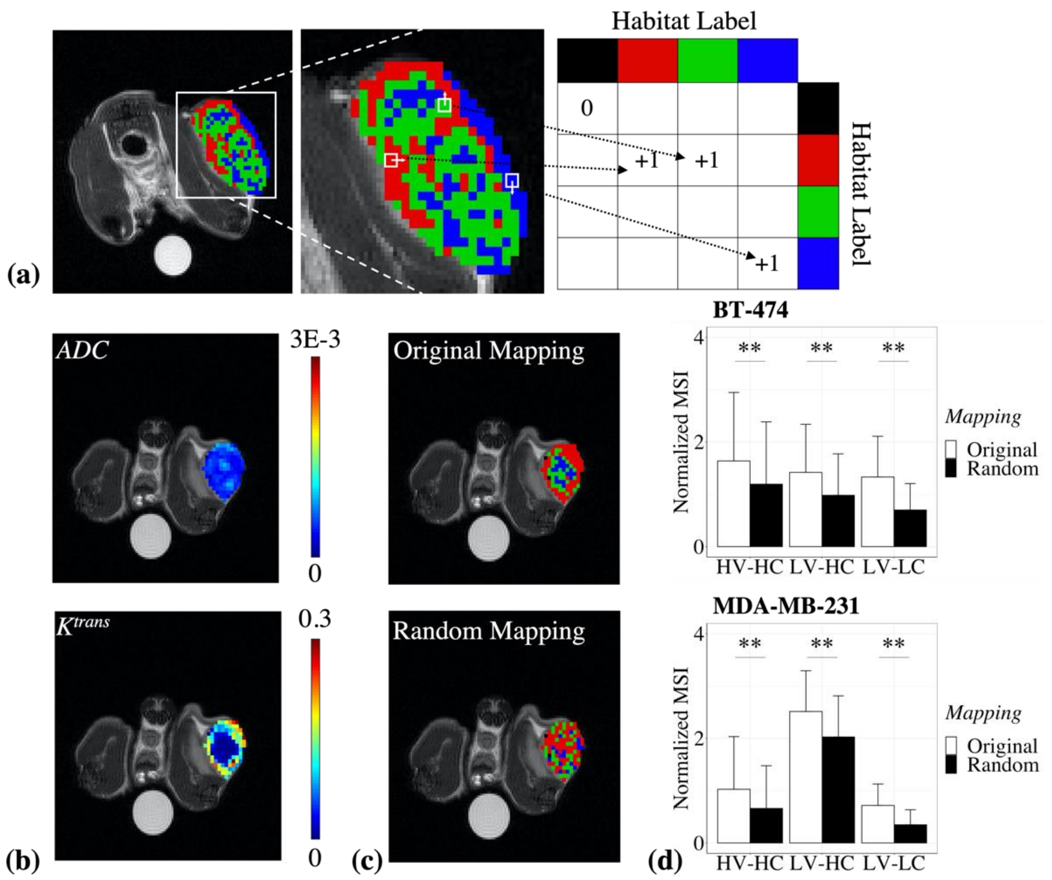

Figure S2. Quantifying spatial colocalization with multiregional spatial interaction (MSI) matrix analysis. An illustration of the MSI matrix [1] is shown in panel (**a**), where the HV-HC habitat is represented in red, LV-HC in green, LV-LC in blue, and non-tumor regions in black. Diagonal values of the MSI matrix represent the connected size of individual habitats and were used to quantify the level of spatial colocalization of habitat voxels. Panel (**b**) shows example tumor *ADC* (mm^2/s) and K^{trans} (min^{-1}) parameter maps. The corresponding habitat map is shown in the top row of panel (**c**) where cluster-labeled voxels were mapped back to their original spatial location ("original mapping"). Once habitat maps were generated, MSI matrix values were calculated for each tumor and normalized to tumor volume. For comparison to original mapping, cluster-labeled voxels were mapped to random spatial locations (bottom row of panel (**c**), "random mapping"), and MSI matrix values were calculated. Panel (**d**) shows the mean normalized MSI for each habitat across all tumors in the BT-474 (top row) and the MDA-MB-231 (bottom row) cohorts. Habitat voxels mapped to their original spatial index show significantly higher MSI values compared to random mapping, indicating significant levels of spatial colocalization. Error bars show standard deviation and ** indicates a $p < 0.01$.

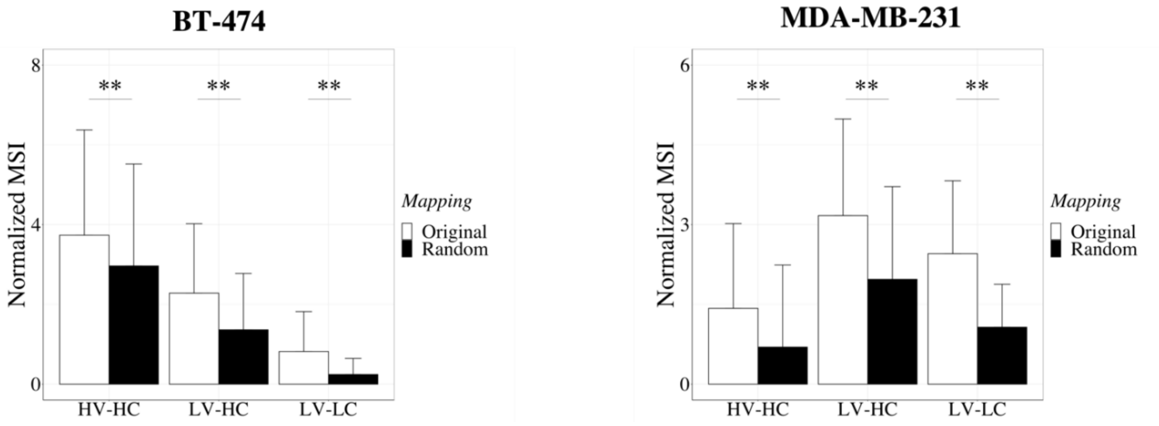

Figure S3. Spatial colocalization of histological habitats was quantified with multiregional spatial interaction (MSI) matrix analysis (see Figure S2). Shown are the mean normalized MSI for each habitat across all tumors in the BT-474 (left) and the MDA-MB-231 (right) cohorts. Histological habitat pixels mapped to their original spatial index show significantly higher MSI values compared to random mapping, indicating significant levels of spatial colocalization. Error bars represent standard deviation and ** indicates a $p < 0.01$.

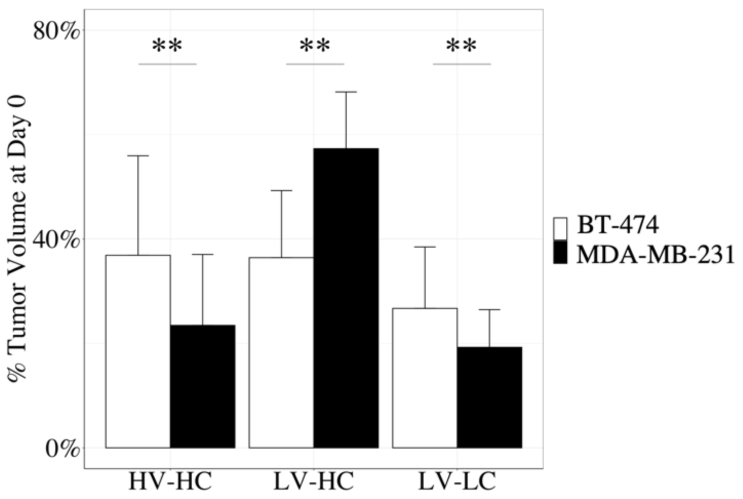

Figure S4. Comparison of baseline tumor composition of BT-474 and MDA-MB-231 tumors in terms of percent tumor volume of each habitat. BT-474 tumors had significantly ($p < 0.01$) lower percent tumor volume of the LV-HC habitat and higher percent tumor volume of HV-HC and LV-LC habitats compared to MDA-MB-231 tumors. ** indicates a $p < 0.01$.

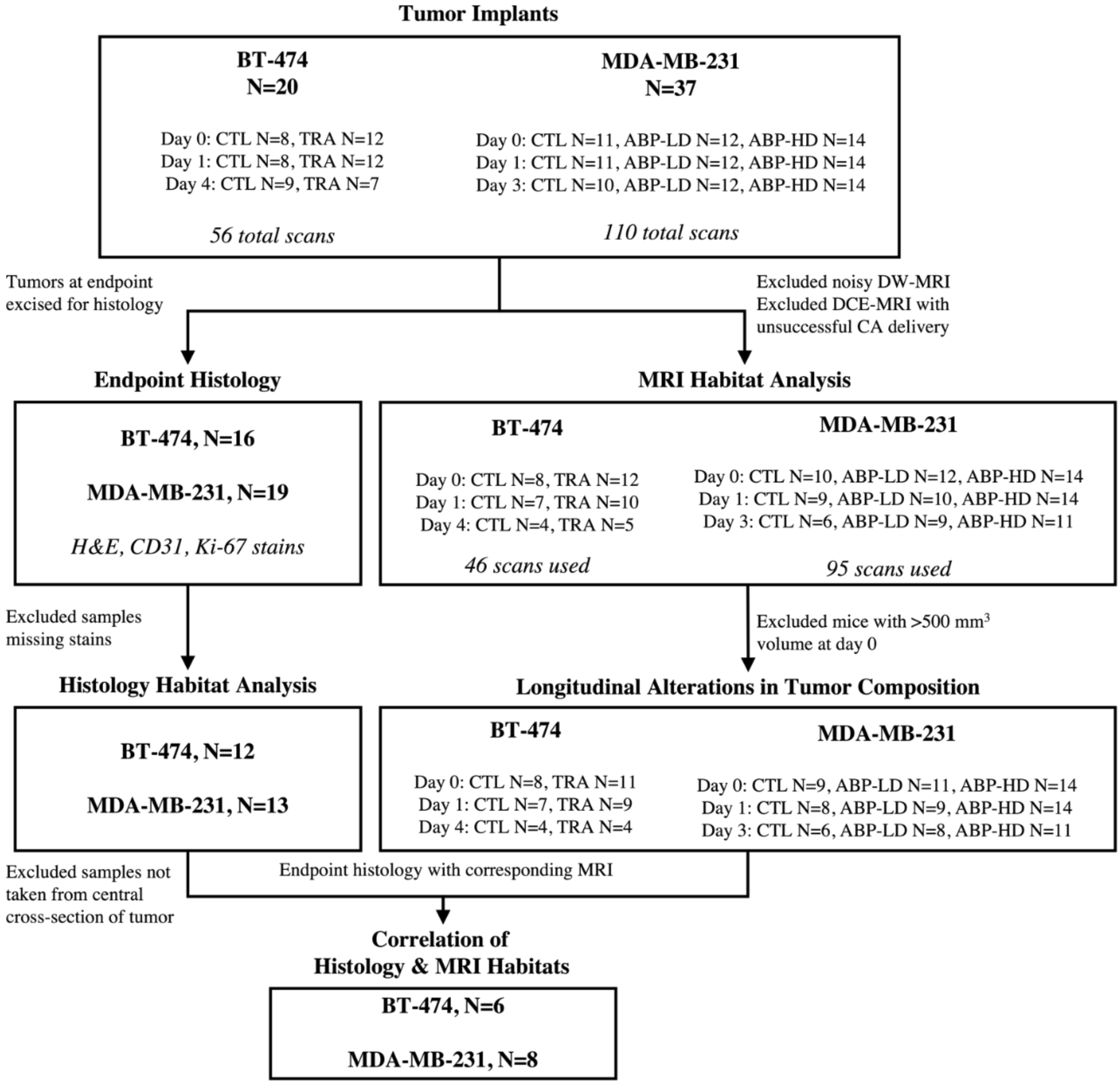

Figure S5. Sample numbers for MRI and histology analysis across cohort treatment groups. The first row shows the number of mice per treatment group imaged at each time point. The left column shows the number of histology sections acquired at the study end point (second row) and the number included in the habitat analysis (third row). The right column shows the number of scans included in the habitat analysis (second row) and the number include in the longitudinal analysis of treatment response (third row). The final row shows the number of mice included in the correlation analysis of imaging and histological habitats. CTL: saline control, TRA: trastuzumab (10 mg/kg), ABP-LD: low-dose albumin-bound paclitaxel (15 mg/kg), ABP-HD: high-dose albumin-bound paclitaxel (25 mg/kg).

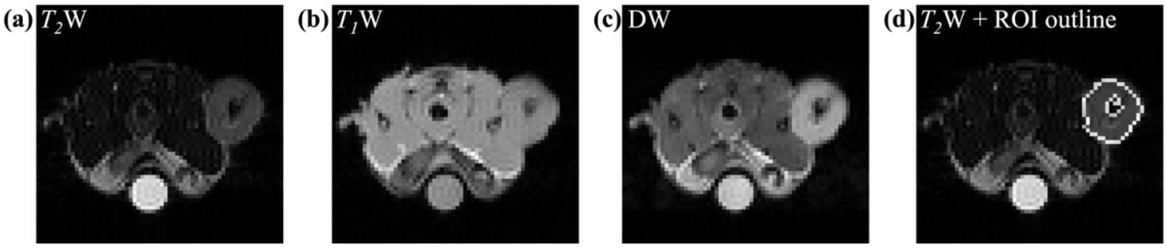

Figure S6. Example T_2-weighted (T_2W; panel (**a**)), T_1-weighted (T_1W; panel (**b**)), diffusion-weighted (DW; panel (**c**) images of an MDA-MB-231 tumor, with the voxels showing signal-voids removed from within the ROI. Panel d (T_2W + ROI outline (d)) shows the outline of the final ROI, which excludes signal-void voxels from analysis. The average (and 95% confidence interval) percent of tumor voxels removed from ROIs was 1.9% (0.29%) per MDA-MB-231 tumor and 0% (0%) per BT-474 tumor.

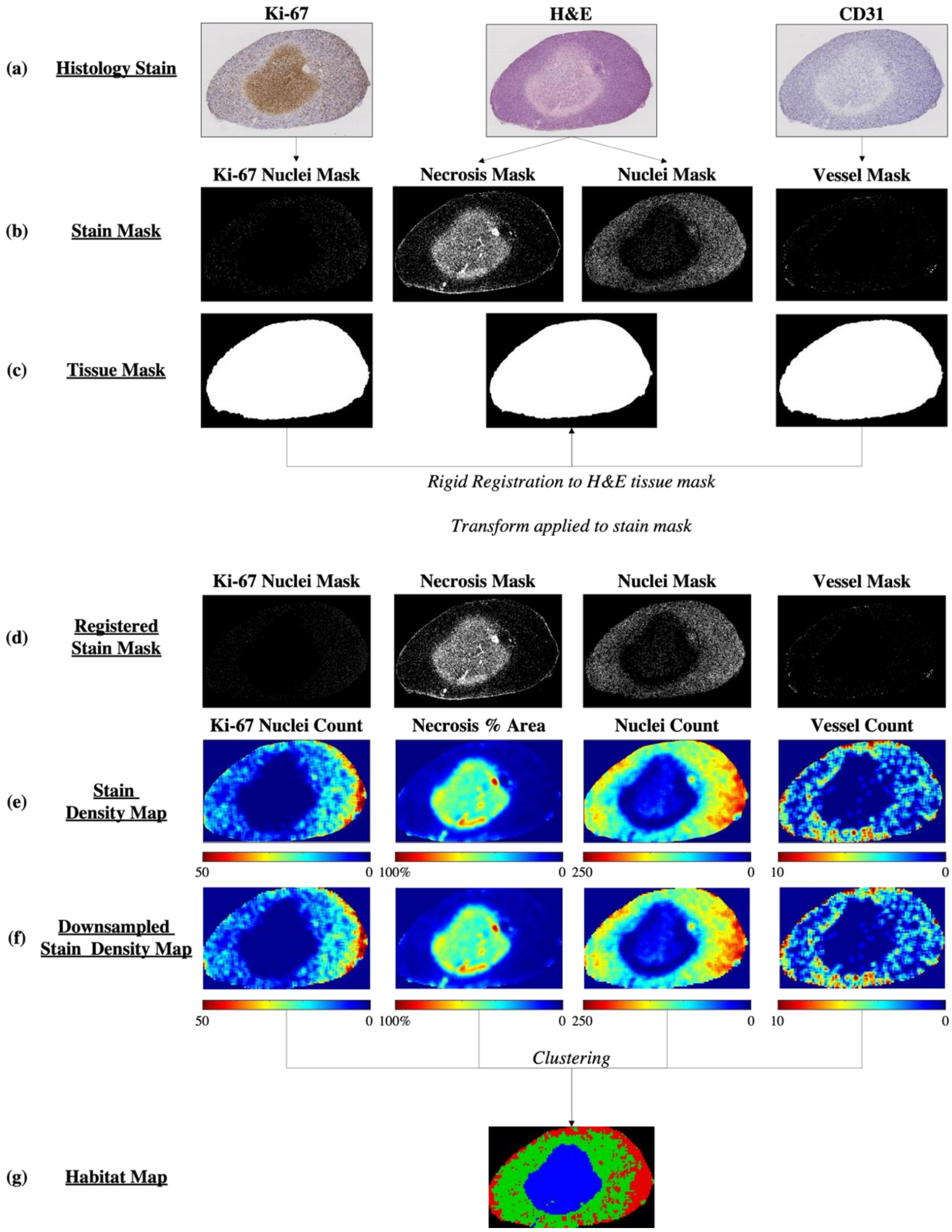

Figure S7. Image processing steps for histology data. Row (**a**) shows three adjacent sections of the same tumor stained with (from left to right) anti-Ki-67, H&E, and anti-CD31. Row (**b**) shows the corresponding stain masks resulting from automated segmentation of (from left to right) Ki-67+ nuclei, necrosis (H&E), nuclei (H&E), and CD31+ microvessels. The corresponding whole tissue masks are shown in Row (**c**), which are used to spatially align the CD31- and Ki-67-stained sections to H&E sections through a rigid registration. The transformation from the rigid registration of the whole tissue masks was then used to register the masks of the CD31 and Ki-67 stain masks to the H&E data (Row (**d**)). A neighborhood analysis was then performed where the number of nuclei (Ki-67+ nuclei, H&E nuclei), necrosis percent area (H&E), or the number of microvessels (CD31) within a 150 μm × 150 μm area around each pixel was quantified and assigned as that pixel's stain density value. Row (**e**) shows stain density maps of (from left to right) Ki-67+ nuclei count, H&E necrosis percent area, H&E nuclei count, and CD31 vessel count. Stain density maps were downsampled by a scale factor of 0.01 (Row (**f**)) and then clustered through agglomerative clustering to yield the final histological habitat map (Row (**g**)). Scale bar

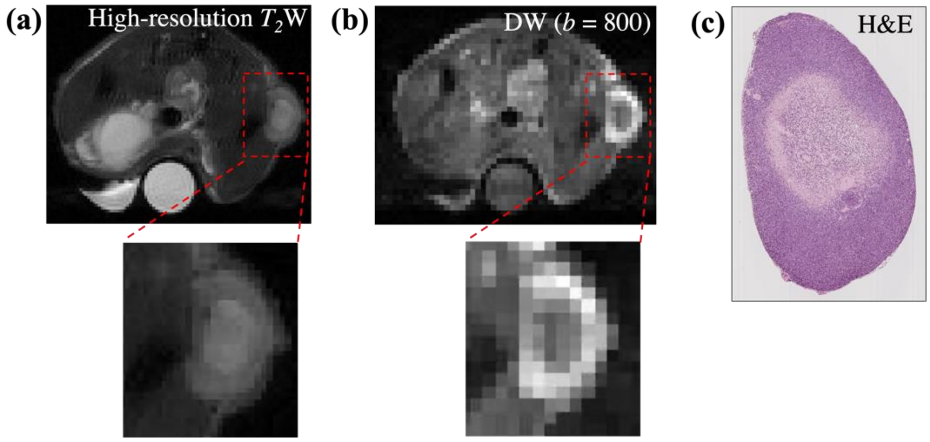

Figure S8. An example central tumor slice from endpoint MRI data and corresponding histological section. To identify central ROI slices, high-resolution T_2-weighted MRI (**a**) and b = 800 DW-MRI data (**b**) were visualized beside corresponding H&E histology sections (**c**). Anatomical similarity of overall tumor slice shape and regions of necrosis was qualitatively evaluated to determine the closest possible central slice match. Scale bar

Supplemental Methods

Agglomerative Clustering

Agglomerative clustering is a hierarchical clustering technique which begins by considering each voxel to be a single cluster. The algorithm then repeatedly merges pairs of clusters that are most similar, as determined by a similarity metric (also known as a "linkage criteria"), until a single cluster is left. Through the clustering process a binary merge tree (dendrogram) is built, showing the hierarchical relationship between clusters.

Quantifying Spatial Colocalization with Multiregional Spatial Interaction (MSI) Matrix Analysis

As no spatial information was used in the clustering process, it is important to determine whether identified clusters spatially resolved into tumor subregions, or "habitats". To determine whether cluster-labeled voxels colocalized spatially, we used a multiregional spatial interaction (MSI) matrix, adapted from work by Wu *et al*. [1], to quantify the level of spatial colocalization of cluster-labeled voxels. For each voxel within a habitat map, each of its surrounding neighbors was evaluated (i.e., 8 neighbors total for each voxel with a 3 × 3 neighborhood). The different combinations of voxel-neighbor pairs (e.g., HV-HC and HV-HC, HV-HC and LV-HC, etc.) were organized as a 4 × 4 matrix, referred to as the MSI matrix (Supplemental Fig. 3a). Each instance of a neighbor-voxel pair was added to the corresponding entry in the MSI matrix. This process was iterated over all tumor voxels until all voxel habitat interactions were quantified to generate a final MSI matrix. The diagonal values of the MSI matrix represent the connected size of individual habitats and were used to quantify the level of spatial colocalization.

Cluster-labeled voxels were mapped to their original spatial index to generate habitat maps and MSI matrices were calculated for each tumor (Supplemental Fig. 3c, top row). For comparison, cluster-labeled voxels were also mapped to a random spatial index and MSI matrices were calculated (Supplemental Fig. 3c, bottom row). The values of the MSI matrix diagonal (HV-HC and HV-HC, LV-HC and LV-HC, LV-LC and LV-LC) were normalized by tumor size. To evaluate whether clustered-voxels colocalized spatially, the normalized MSI values for the original mapping were compared to

that of the random mapping, using the pairwise Wilcoxon rank sum test, with a p-value less than 0.05 considered significant.

References.

1. Wu, J.; Cao, G.; Sun, X.; Lee, J.; Rubin, D.L.; Napel, S.; Kurian, A.W.; Daniel, B.L.; Li, R. Intratumoral Spatial Heterogeneity at Perfusion MR Imaging Predicts Recurrence-free Survival in Locally Advanced Breast Cancer Treated with Neoadjuvant Chemotherapy. *Radiology* **2018**, 172462, doi:10.1148/radiol.2018172462.

© 2020 by the authors. Licensee MDPI, Basel, Switzerland. This article is an open access article distributed under the terms and conditions of the Creative Commons Attribution (CC BY) license (http://creativecommons.org/licenses/by/4.0/).
